# 

**Published:** 1904-08-15

**Authors:** 


### CONTENTS.

The Fourth International Dental Congress,

*By Edward C. Kirk, D.D.S., SC.D.* 379

Suggestions in Dental Records, Book-keeping and Nomenclature, - - - *By H. T. Smith, D.D.S.* 385

The Doctor Analyzed, - *By J. W. Clemmer, M.D.* 393

Program of the Fourth International Dental Congress - 408

Editorial, - - - - - - - - 415

Announcements, - - - - - - - - 418

Obituary, - - - - - - - - 422

Indents, - - - - - - - - - 423

